# Cutaneous neurogenic inflammation in the sensitized acupoints induced by gastric mucosal injury in rats

**DOI:** 10.1186/s12906-017-1580-z

**Published:** 2017-03-07

**Authors:** Wei He, Xiao-Yu Wang, Hong Shi, Wan-Zhu Bai, Bin Cheng, Yang-Shuai Su, Xiao-Chun Yu, Xiang-Hong Jing, Bing Zhu

**Affiliations:** 1 Institute of Acupuncture and Moxibustion, China Academy of Chinese Medical Sciences, Nanxiaojie 16, Dongzhimennei, Beijing, 100700 China; 2 The Affiliated Hospital, Shandong University of Traditional Chinese Medicine, No. 42, Wenhuaxi Road, Jinan, 250014 China

**Keywords:** Sensitized acupoints, Gastric mucosal injury, Nociceptive neuropeptide, HA, 5-HT, Mast cell

## Abstract

**Background:**

In acupuncture practice, the most important step is to confirm the location of a sensitized acupoint which reflects a diagnosis and can be stimulated with a specialized needle to treat the disease. Abnormal symptoms such as hyperalgesia or allodynia at the sensitized acupoints in patients with visceral disorders are considered to be in relation with referred pain and neurogenic inflammation. Yet, limited study has investigated the cutaneous neurochemical changes of the sensitized acuponits.

**Methods:**

The resent study developed an animal model of gastric mucosal injury (GMI) by HCl administered into the stomach of the rats. Evans Blue (EB) dye was applied by injection of tail vein after mucosal damage to observe the neurogenic plasma extravasation dots in the skin of the rats. The EB dots extravagated in the skin were compared with locations of acupoints. Immnohistochemistry analysis was used to detect the expression of calcitonin gene-related peptide (CGRP)- or substance P (SP)-labeled nerve fibers, histamine (HA)-, serotonin (5-HT)-, and tryptase-labeled cells in EB dots. Images were recorded and analyzed by Confocal imaging system and Olympus Image Processing Software.

**Results:**

The results showed that GMI resulted in neurogenic plasma extravasation in the skin of the acupoints over the back and abdomen, which mostly occurred in the T9-11 dermatomere. The EB extravasation dots appeared after GMI and disappeared gradually during the natural self-recovery of the gastric mucosa. More SP and CGRP positive nerve fibers were distributed in EB dots than that in regions beside EB dots and in the control, mostly distributed in the nerve fibers around both the vessels and root of hair follicle. Mast cells also aggregated and degranulated to release algogenic substances of 5-HT and HA around the vessels in areas of the EB dots.

**Conclusions:**

Our results indicates that the mechanism of EB extravasation in the skin of the acupoints induced by GMI are closely related to neurogenic inflammation, and that the high expression of local allergic substances and nociceptive neuropeptides in the local skin including SP, CGRP, HA, 5-HT, and mast cell tryptase may be the underlying mechanism of the acupoint sensitization.

## Background

In Chinese acupuncture practice, doctors’ careful detection of acupoints and patients’ subjective response to acupoint stimulation are major factors in effective treatment. Clinically, the most important step is to confirm the location of a sensitized acupoint, which then reflects a diagnosis, and can be stimulated with a specialized needle for treating the disease. Abnormal symptoms such as hyperalgesia or allodynia occurred at some sensitized acupoints in most patients when they are suffered with visceral disorders [1]. The sensitized points may be regular points, extra points, or “Ashi” points [2].

Hyperalgesia or allodynia at the sensitized acupoints are considered to be in relation with “referred pain”, a term defined by Head in 1983 and refers to the many forms of visceral pain felt in regions of the body other than the organ whose stimulation causes pain [3]. Over the past three decades connections between Head zones and acupoints have been discussed [4, 5]. A comparison of Head’s papers with the oldest still extant Chinese sources on acupuncture revealed astonishing parallels between the two concepts regarding both point locations and functional aspects [4]. Strong functional relations between all Head zones, channels, and acupoints were found when following the pattern of segmental dermatomes [5], that is, areas of skin innervated by one and the same spinal nerve.

Previous studies had suggested that mechanism of the referred visceral pain is related to central sensitization of the spinal cord and neurogenic inflammation [6, 7]. Recently,animal study on the mechanism of acupoint sensitization confirmed that pathological change of internal organs’ functional activity lead to the sensitization of spinal center and further to the changes of the size and function of acupoints on body surfaces [8]. Yet, limited study has investigated the cutaneous neurochemical changes of the sensitized acuponits.

The purpose of this study was to develop an animal model of gastric mucosal injury (GMI) by acid-induced nociceptive event in rats. Evans Blue (EB) dye was applied by injection of tail vein after mucosal damage and the referred EB dots extravagated in the skin [7] were observed to give evidence for the kinetic distribution of sensitized acupoints. Immunohistochemistry analyses were used to observe the changes of mast cells (MCs), the expression of nociceptive neuropeptides calcitonin gene-related peptide (CGRP) and substance P (SP), and allergic substances such as histamine (HA) and serotonin (5-HT) in the skin in the extravasated EB dots, to further investigate the underlying mechanisms.

## Methods

### Animals and model procedures

All experimental protocols reported here were in accordance with the National Institute of Health Guide for the Care and Use of Laboratory Animals (NIH Publications No. 80–23) revised 1996 and the Animal Use and Care of Medical Laboratory Animals from the Ministry of Public Health of People’s Republic of China. All efforts were made to minimize the number of animals used and their suffering. Sprague-Dawley rats, weighing 180–220 g, were bought from the institute of Zoology, Chinese Academy of Sciences. The rats were housed in a room maintained at 24 ± 1 °C and illuminated for 12 h (07:00 to 19:00) every day, and fasted for 20 h prior to experiments but had free access to water. Thirty-six rats were randomly divided into six groups: HCl 1d group, HCl 2d group, HCl 3d group, HCl 4d group, HCl 5d group, and Saline control group, with 6 rats in each group. Hydrochloric acid (HCl, 0.5 M) or saline (as a control) was administered orally at a volume of 1 mL/100 g via a soft pediatric feeding tube [9]. After modeling, thirty rats in all the HCl groups were used to observe the dynamic change of EB extravasated points in successive five days. After observation the EB extravasated points, the stomachs and the skin of the six rats in the HCl 1d group were used for immunochemistry experiments.

### Evans blue dye injection and assessment of cutaneous plasma extravasation

Five to six hours after HCl administration, the rats were anesthetized with pentobarbital (50 mg/kg i.p.). EB Dye (Sigma Chemical Co, St. Louis, MO) dissolved in sterile water (50 mg/kg) was then injected through the tail vein. Skin color changes were observed for 1–2 h, and the area of dye extravasation was sketched on the body charts and, in some rats, photographed. Care was taken not to cause any injury to the skin before and during hair removal. The hair of the back, thorax, abdomen, arms, and legs was shaved and sometimes completely removed using commercially available hair removal cream.

### Tissue preparation and immunohistochemical staining

After dye extravasation was sketched, rats were fixed with 0.1 M phosphate-buffered saline (PBS) and 4% paraformaldehyde via transcardial perfusion before removing the area of skin with the extravasated EB. Then the EB extravasated skin and its control area within a 2 mm radius from the extravasated dots, as well as the corresponding skin areas in saline treated rats, were collected. Following perfusion–fixation, the collected tissues were post-fixed in 4% paraformaldehyde at 4 °C for 4 h and cryoprotected in phosphate-buffered 20% sucrose at 4 °C for 24 h. Following post-fixation, the skin was embedded in artificial medium (Shandon Cryomatrix™, 120 mL, Thermo Scientific, USA), frozen, and cut into 30 μm sections. The sections were then thaw-mounted on SuperFrost® Plus slides (Thermo Scientific, USA) and allowed to dry. Skin sections from HCl- and saline-treated rats were mounted on the same slide to guarantee equal incubation conditions. After an initial wash in 0.1 M PBS (pH 7.4) tissues were preincubated in a solution of 3% normal goat serum and 0.5% Triton X-100 in 0.1 M phosphate-buffered solution (PB, pH 7.4) for 30 min to block non-specific binding. Antibodies were diluted in a solution containing the same substances. Sections were then incubated for 24 h at 4 °C. Immune-fluorescent staining for CGRP, SP, 5-HT or HA was conducted by using specific primary antibodies (Table 1) from different species, mouse and rabbit, which were incubated with the sections simultaneously. After washing in 0.1 M PBS (3–10 min), tissue was exposed to Alex a Fluor 488 goat anti-mouse (1:500; A11001, 0.5 mL, Invitrogen, USA) or Alexa Fluor 488 goat anti-rabbit (1:500; A11008, 0.5 mL, Invitrogen, USA) IgG secondary antibody for 2 h and washed with 0.1MPB. The tissue was then counterstained with blue-fluorescent DAPI nuclei acid (1/40,000, D3571, 10 mg, Invitrogen, USA) for 5 min to label cell nuclei. Following a final wash in 0.1 M PBS, slides were coverslipped with PBS–glycerol. Negative controls were performed by leaving out the primary antibodies during the staining procedure. Mast cells were labeled with the anti-mast cell tryptase antibody and, simultaneously, by HA or 5-HT primary antibodies to observe the coexpression, then were also counterstained with DAPI for nucleus labeling. The secondary antibodies were Alexa Fluor 488 goat anti-rabbit IgG secondary antibody (c; A11008, 0.5 mL, Invitrogen, USA) or Alexa Fluor 594 goat anti-mouse IgG secondary antibody (1:500; A11005, 0.5 mL, Invitrogen, USA). Slides were recorded with confocal imaging system (FV1200, Olympus, Japan) and analyzed by the Olympus Image Processing Software by an investigator who was blinded to group. Approximately 20 randomized sections from each group were counted for the number of stained cells, the sum length of positive nerve fibers, and the sum fluorescent intensity. All immunohistochemistry for each staining combination was performed at the same time to ensure staining consistency.

**Table 1:** Primary antibodies for immunohistochemistry

Antibody	Species	Dilution	Source	Product no.	Company
Anti-CGRP Anti-Substance P Anti-Serotonin Anti-Histamine Anti-Mast Cell Tryptase	Rat Rat Rat Rat Rat	1/1000 1/1000 1/500 1/500 1/1000	Mouse Rabbit Rabbit Rabbit Mouse	ab81887 ab48326 ab10385 ab123982 ab2378	Abcam Abcam Abcam Abcam Abcam

### Data analysis and statistical analysis

EB dye extravasation was quantified by counting the number of blue dots in the skin over the same dermatomere areas. Data are expressed as mean ± SEM. The Kolmogorov-Smirnof test was used to evaluate if these groups fit the normal distributions. Normally distributed groups were analyzed by parametric tests. Comparison between three groups was made using the One-Way ANOVA test followed by the *LSD* or *Dunnett’s T3* post-hoc test. Nonnormally distributed groups were analyzed by the Mann-Whitney test. *P* < 0.05 was considered significant.

## Results

### GMI induced EB extravasated points in certain acupoints

**Fig. 1:**
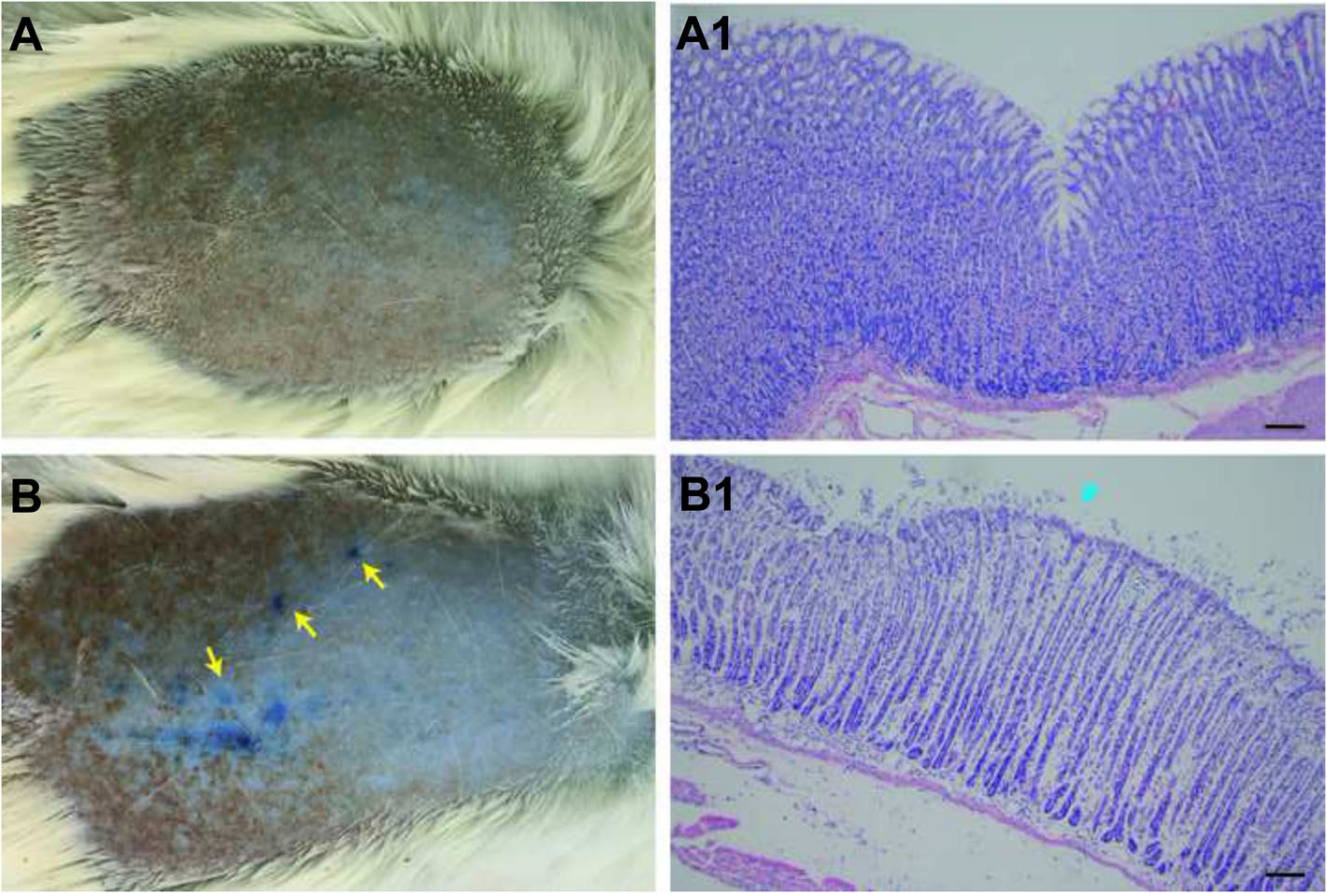
EB extravasated points in the skin and histological examination of the gastric mucosa in rats. Images of **a**, **a1** were obtained from the control group, images of **b**, **b1** were obtained from the HCl 1d model group. Six hours post-HCl administration into the stomach of the rats, EB extravasated points (*yellow arrow*) in the model group (**b**) increased in comparison with that in the control group (**a**). Histological examination showed damaged epithelial cells including epithelial cell loss, edema, and the presence of inflammatory cells (**b1**, *Blue arrow*) in the model group. The scale bar is 50 μm

In rats that received HCl administration, small blue extravasation EB dots or areas appeared in the skin over the back and abdomen, especially prominent over the back (Fig. 1B, yellow arrow). While, no cutaneous plasma extravasation was observed in 3 of the saline-control rats that received saline administration into the stomach (Fig. 1A). Six hours after HCl administration, the stomach of the rats has been histological examined, which showed that administration of HCl into the stomach resulted in the damage of gastric mucosa of the rat, which included epithelial cell loss, edema, and the presence of inflammatory cells (Fig. 1B1, blue arrow).

**Fig. 2:**
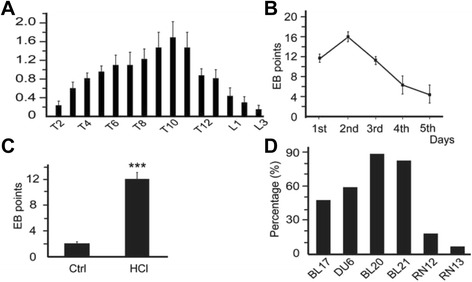
Distribution of EB extravasated points. **a** Segmental distribution of EB extravasated points and their concentration in the T6-12 region of the dermatomere. **b** Kinetic appearance of the EB dots throughout the 5d and their gradual disappearance as times go on. **c** Comparison of the mean numbers of the EB dots per rat in the skin in experimental rats after GMI. (*** *P* < 0.001 *vs* Ctrl). **d** The relevancy rate between the EB points and the acupoints

The number and distribution of blue dots varied considerably between rats, but they concentrated in the T6-12 dermatomere region (Fig. 2a). The dots started to appear about 10 min following the injection of EB, ranged in size from 0.5 to 2 mm in diameter, and the majority of dots were visible within 30 min, and rarely, additional dots or patches appeared later. The dots appeared with GMI and disappeared gradually in five days (Fig. 2b). To quantify the extent of neurogenic plasma extravasation in the skin, the number of blue dots was recorded. The mean number of small blue dots per rat significantly increased in HCl-treated rats as compared to the control rats (*P* < 0.001) (Fig. 2c). The minimal cutaneous extravasation in the 3 other saline-treated rats may be associated with the abdominal injection of pentobarbital or minimal injury caused by the hair removal cream. Most of the blue dots distributed over the back skin and appeared close to the midline. When the locations of EB extravasation points were compared with those of regular acupoints, the blue dots corresponded almost identically to acupoints. The percentage of the correlation between the acupoints and the EB points were BL17:47.5%, DU6:58.82%, BL20:88.23%, BL21:82.35%, RN12:17.64% and RN13:5.88% (Fig. 2d).

### Nociceptive neuropeptides CGRP and SP were highly expressed in EB extravasated dots

**Fig. 3:**
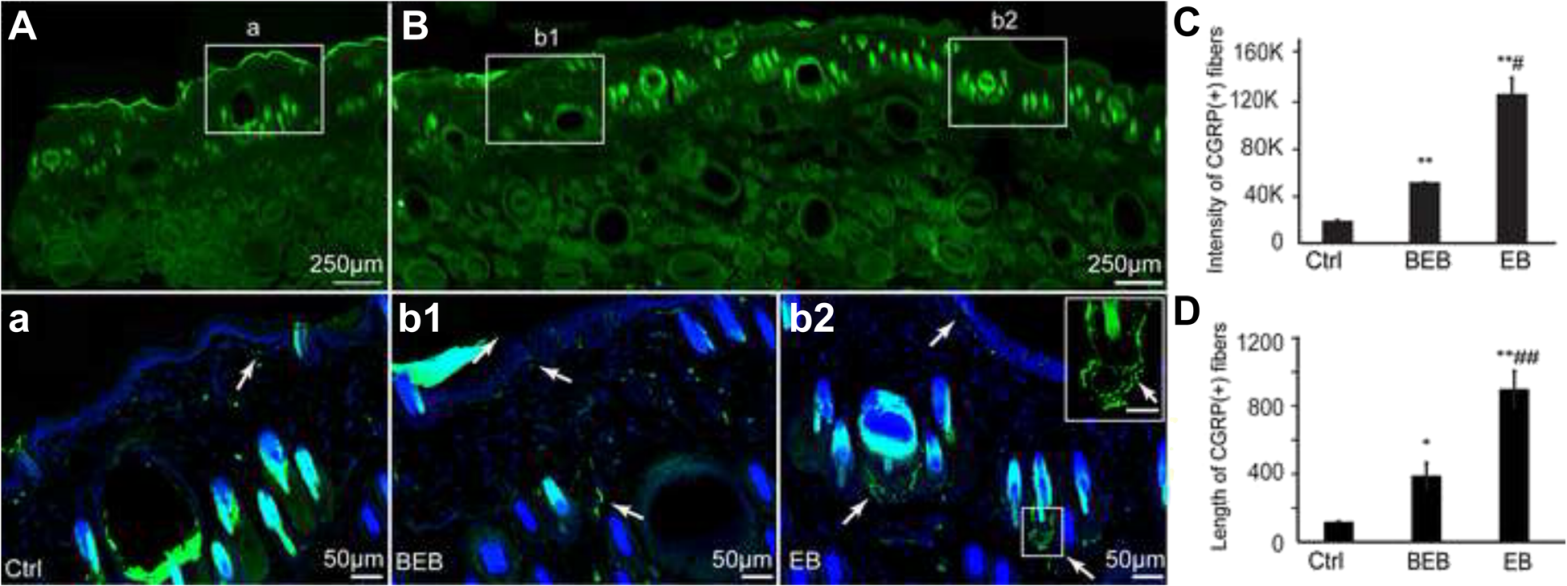
Distribution of CGRP immune-positive fibers in extravasated dots, beside EB, and in saline control. Representative immnuofluoresence staining images of CGRP in each group was shown **a** and **b**. The corresponding images below were enlargements of the boxes outlined in **a** and **b**. CGRP (+) fibers were expressed mainly in the epidermis and dermis (*white boxes*). **a** Few CGRP (+) fibers in dermis of skin in saline-control (*white box*). **b** Distributions of CGRP (+) fibers in extravasated EB dots (b2) and beside EB dots (b1). Note that the CGRP (+) nerve fibers in EB dot were more highly expressed than that in BEB and the control (*white arrow*). The box in b2 show the CGRP (+) nerve fibers in the EB dot. The right corner magnification box display CGRP (+) fibers that formed a neural net around the root of the hair follicle (Scale bar: 20 μm). **c**, **d**. Sum fluorescent intensity and sum length (μm) of CGRP (+) nerve fibers per section were significantly higher in EB than in the control and in BEB (*P < 0.05 *vs* Control; ** *P* < 0.01 *vs* Control; # *P* < 0.05 *vs BEB*; ## *P* < 0.01 *vs BEB*)

**Fig 4:**
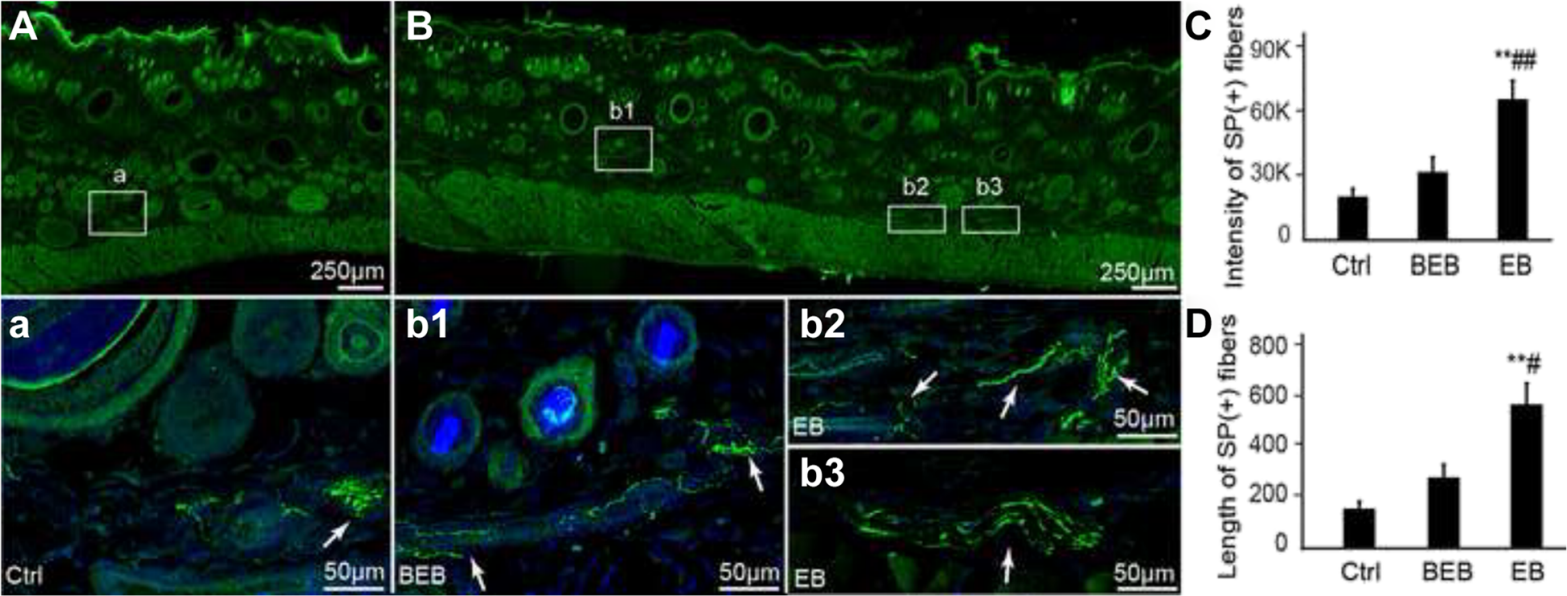
Distribution of SP immune-positive fibers in extravasated dots, beside EB, and in saline control. **a**, **b**. Representative immnuofluoresence staining images of SP in each group. SP (+) fibers are expressed mainly in the dermis (*white boxes*). **a** The few SP (+) fibers in dermis of skin in saline-control (*white box*). The corresponding images below were enlargements of the boxes outlined in **a** and **b**, and showed the nerve fibers of SP (+) (*white arrow*). **b** Distribution of SP (+) fibers in EB extravasated dots (b2 and b3) and beside EB dots (b1). Note the box was the location of SP (+) nerve fibers. SP labelled nerve fibers in EB dots were distributed more densely under the dermis than they were around the EB dots and in the control. b2 and b3 displayed SP (+) fibers forming a nerve plexus in the dermis. **c**, **d**. Sum fluorescence intensity and sum length (μm) of SP (+) fibers per section were expressed significantly more in EB than in the control and BEB (** *P* < 0.01 *vs* Control; # *P* < 0.05, *vs BEB*; ## *P* < 0.01, *vs BEB*)

The locations of neuropeptides of CGRP and SP labeled nerve fibers were determined by immnohistochemical examination. CGRP positive nerve fibers were detected in the epidermis, around the roots of hair follicles, and near the vessels, which showed elevated expression in and around the EB dots compared to the control (Fig. 3). Some CGRP positive fibers formed a neural net around the roots of the hair follicles (Fig. 3b2). Moreover SP positive nerve fibers were also detected in the dermis where EB dots were present, and these fibers distributed more densely under the dermis than they did around the EB dots and in the control (Fig. 4). In conclusion, both CGRP and SP were highly expressed in EB dots induced by GMI. The sum fluorescent intensity and the length of both CGRP (+) and SP (+) fibers were expressed significantly more in EB dots than in areas around EB dots and in the saline control (Fig. 3C and D, Fig. 4C and D).

### Mast cells aggregated and degranulated and released HA and 5-HT in EB extravasated dots

**Fig. 5:**
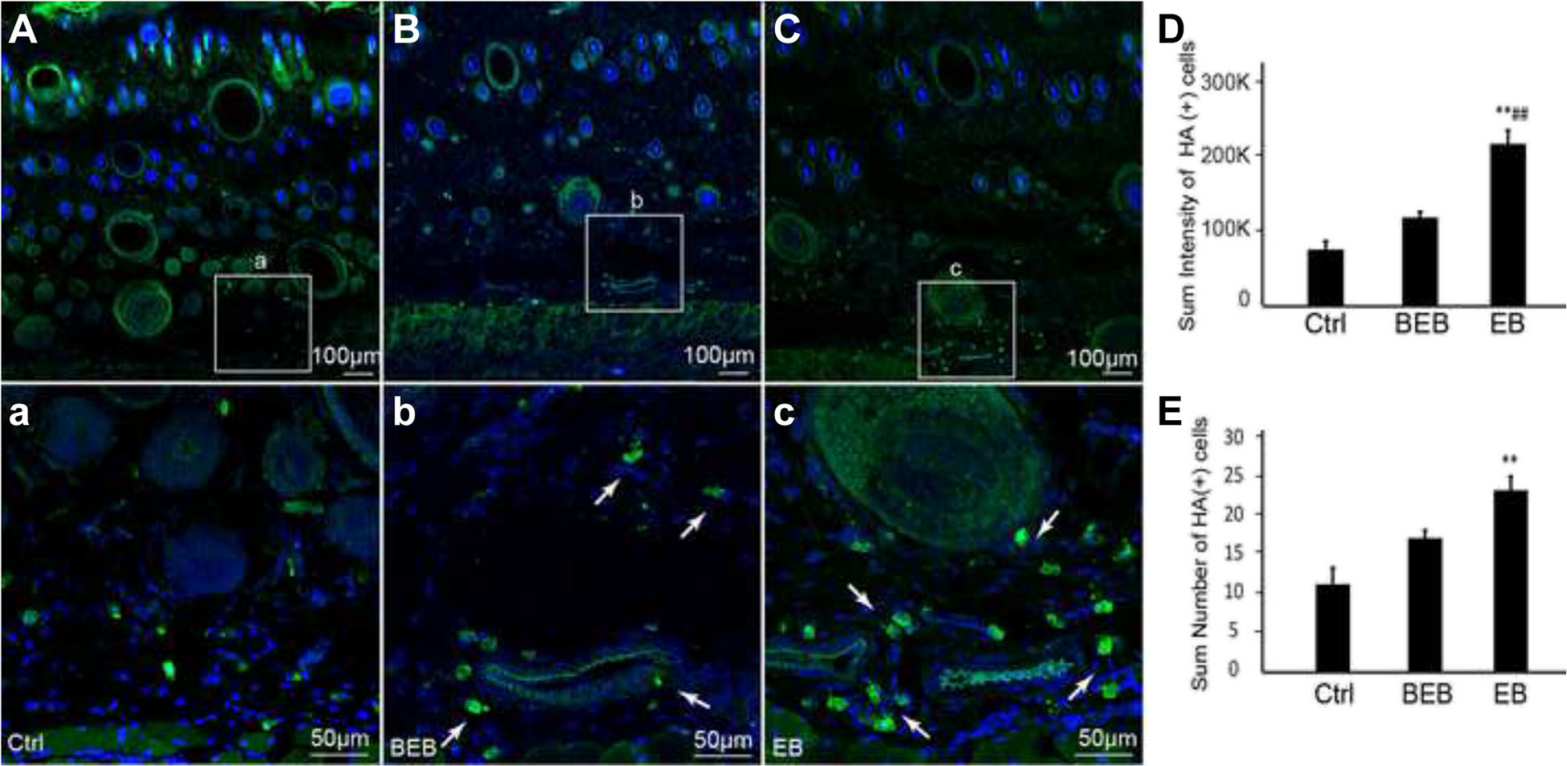
Distribution of HA immune-positive cells in extravasated dots, beside EB, and saline control. **a**, **b**, **c**. Representative immnuofluoresence staining images of HA in each group. There were very few HA labelled cells in dermis of saline-control (**a**) and BEB (**b**). However, HA labelled cells in EB dots were expressed more in EB than in BEB and the control (**c**). The corresponding images below were enlargements of the boxes outlined in **a**, **b** and **c**. Note that the labelled cells were located near the vessels. D, E. The sum fluorescent intensity and the number of HA (+) cells per section were significantly higher in EB than in the control and BEB (** *P* < 0.01 *vs* Control; ## *P* < 0.01, *vs* BEB)

**Fig. 6:**
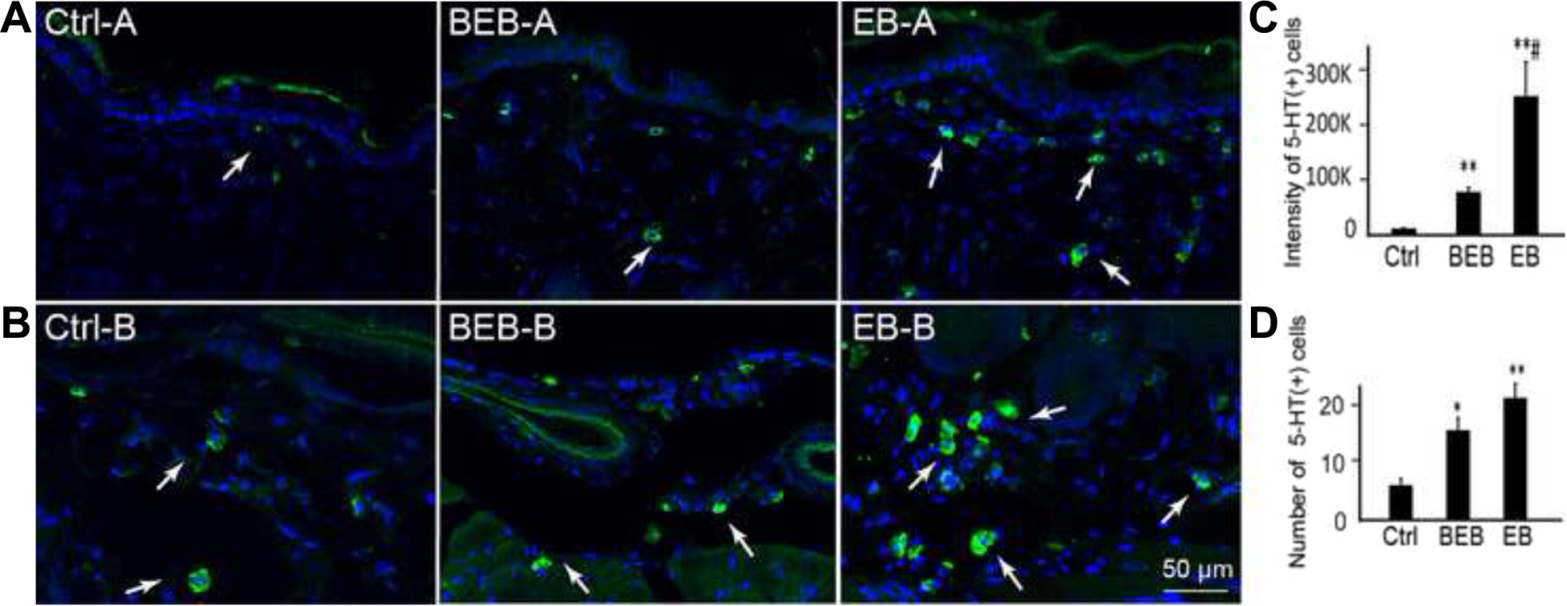
Distribution of 5-HT immune-positive cells in extravasated dots, beside EB, and in saline control. **a**, **b**. Representative immunofluorescence staining images of 5-HT under the epidermis (in *green*, A row) and in the dermis (in *green*, B row). There were a few 5-HT labelled cells under the epidermis (*White arrow*) and some 5-HT (+) cells in the dermis (White arrow). **c**, **d**. The sum fluorescent intensity and number of 5-HT (+) cells of per section were significantly higher in EB than in the control and BEB (* *P* < 0.05 *vs* Control; ** *P* < 0.01 *vs* Control; # *P* < 0.05, *vs* BEB). The 5-HT labelled cells in dermis were located near the vessels

**Fig. 7:**
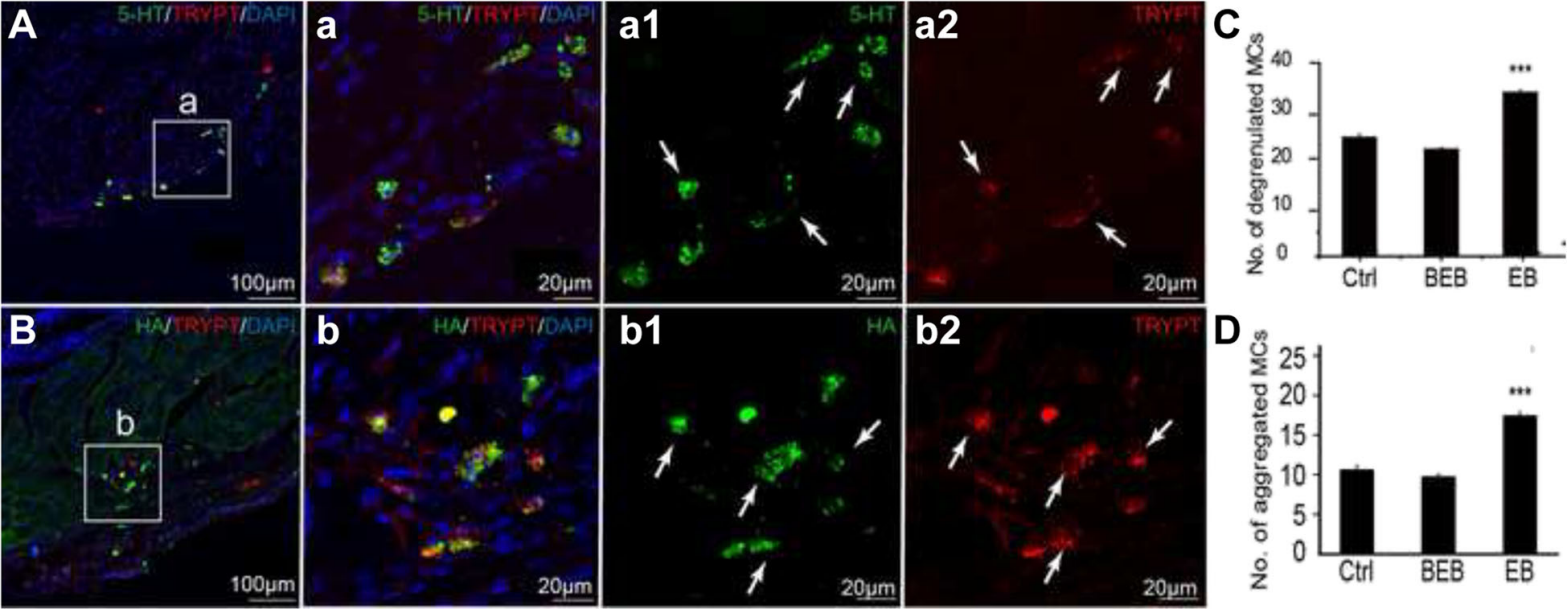
Distribution of 5-HT or HA co-expressed with tryptase (+) mast cells in EB extravasated dots. **a**, **b**. Representative triple-labeling immnuofluoresence staining images of 5-HT or HA, tryptase and DAPI. Triple-labeling immunofluorescent staining of mast cells was performed with either anti-5-HT (A, a, a1 in green) or anti-HA (B, b, b1 in *green*) and anti-tryptase (A, a, a2, B, b, b2, in *red*). The nuclei were labeled by fluorescent DAPI (in *blue*). Triple-labeled mast cells in the dermis co-expressed 5-HT and tryptase or HA and tryptase (*white arrow*). The mast cells aggregate (B, b) and degranulate (a2, b2 arrow) with 5-HT (+) or HA (+) granules (a1, b1). C, D. The number of aggregated and degranulated mast cells were markedly increased in EB dots than in beside EB dots and the control (*** *P* < 0.001 *vs* BEB and Control)

Both HA (+) and 5-HT (+) cells in EB extravasated dots were expressed more in the dermal than around the EB dots and in the saline control (Figs. 5 and 6). Further anti-mast cell tryptase antibody was used to determine the types of these immunoreactive cells, in order to indicate the activation of mast cells during neurogenic inflammation [10]. Mast cell aggregation and degranulation was seen more aggressively in EB dots than in areas around the EB dots and in the saline control (Fig. 7). Mast cells expressed either 5-HT or HA in the dermis, and some of them degranulated 5-HT (+) or HA (+) granules (Fig. 7). In addition, 5-HT labelled cells were also distributed under the epidermis (Fig. 6, white arrow) which were not labelled by anti-mast cell tryptase antibody. The fluorescence intensity and the number of the HA or 5-HT immunoreactive cells per section were significantly higher in the extravasated EB dots than in areas around EB dots and in the saline control (Figs. 5 and 6).

## Discussion

The results of the present study demonstrated that GMI resulted in neurogenic plasma extravasation in the skin of the acupoints over the back and abdomen, mostly in the T9-11 dermatomere. The EB extravasation dots appeared after GMI and disappeared gradually during the natural self-recovery of the gastric mucosa. More SP and CGRP positive nerve fibers were distributed in EB dots than in regions beside EB dots and in the control. Mast cells also aggregated and degranulated to release algogenic substances of 5-HT and HA in the EB dots. Our results indicates that the mechanism of EB extravasation in the skin of the acupoints are closely related to neurogenic inflammation induced by GMI and that the high expression of local allergic substances and nociceptive neuropeptides may be the underlying mechanism of acupoints sensitization.

### Relationship between segmental distribution of EB dots and acupoints

Some of the cutaneous EB dots appeared in the exact locations of acupoints of BL20, BL21, DU6, BL17, RN12, and RN13. These acupoints are frequently used to treat gastrointestinal diseases in Chinese medicine [11, 12]. The EB dots appeared after GMI, and disappeared gradually during the natural self-recovery of the gastric mucosa, which indicated that the local neurochemical substances of the EB dots changed with the different degree of GMI. When the gastric mucosa was injured, most homosegmental acupoints were also “sensitized” and in an active state to reflect the disease, whereas, when the GMI subsided the function of these acupoints recovered to their latent state gradually. The distribution of the sensitized acupoints after GMI was restricted to the dermatomes of T9-11 in the same somite with gastry, which indicated that acupoints in the same somite of suffered viscera tended to be sensitized more than other acupints.

### Neuronal pathways mediating referred pain in the skin of rats after GMI

**Fig. 8:**
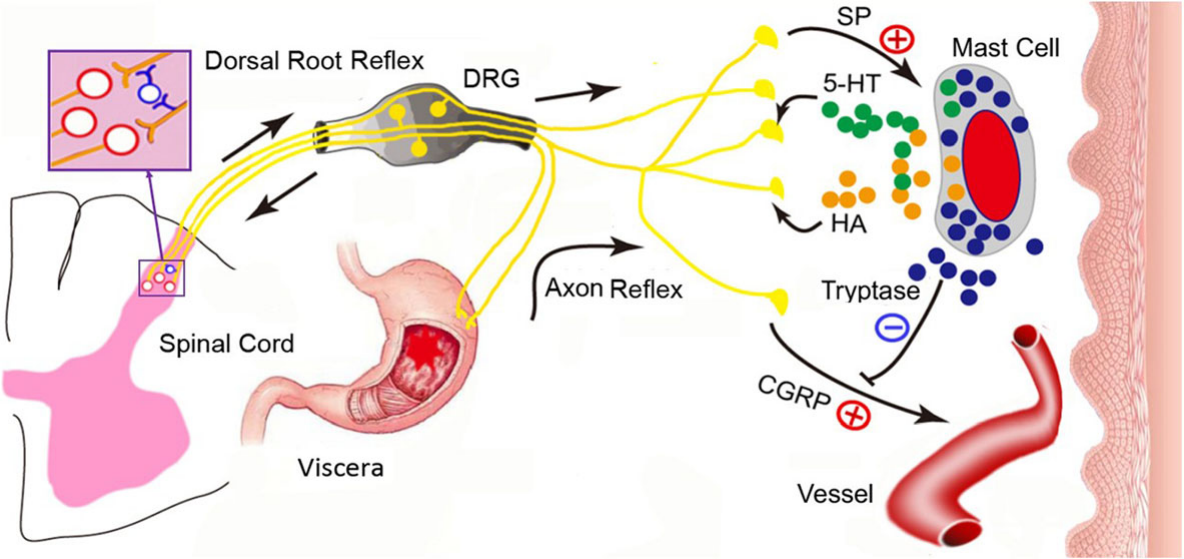
Sketch map of the mechanism of acupoint sensitization due to GMI. 1) Nociceptive information from the gastric mucosa activate neurons in the DRG, and then the impulses travel antidromically toward the periphery via axon reflexes (ARs). 2) Nociceptive information is transmitted orthodromically to the dichotomized neurons in the spinal cord leading to antidromic activation of the somatic branch, or to the neighboring primary afferent fiber through an interneuron via dorsal root reflexes (DRRs). 3) The antidromic activity induced by ARs or DRRs results in the release of inflammatory mediators such as SP and CGRP in skin nerve fibers, resulting in mast cells activation. 4) Activated mast cells release some algogenic substances HA and 5-HT and cause hyperalgesia in local acupoints. 5) In local acupoints, increased SP and CGRP cause plasma extravasation and vasodilatation. SP can also induce aggregation and degranulation of mast cells to release allergic substances HA and 5-HT. All aggravate the neurogenic inflammation further in the local point. This is possible the pathway of acupoint sensitization

Mechanism of referred pain in the skin of rats to visceral injury was considered through the somatovisceral reflex pathway [7, 13]. The neuronal pathway can be potentially explained by the following processes: 1) the gastric nociceptive information is transmitted to the dorsal root ganglion (DRG) and antidromically to the periphery afferent fibers via axon reflex (AR), with a result of antidromic activation of afferent fibers, 2) the nociceptive information is transmitted orthodromically to the dichotomized neurons in the spinal cord leading to antidromic activation of the somatic branch [14], or the nociceptive information antidromically activate the neighboring primary afferent fiber through a interneuron in the spinal cord via dorsal root reflexes (DRRs), resulting in neurogenic plasma extravasation and induction of neurogenic inflammation in the related dermatomes [15]. 3) this antidromic DRRs and AR result in the release of neuropeptides (e.g., SP and CGRP) from both C and Aδ fibers [16, 17]. SP as well as other tachykinins acts on NK receptors to increase microvascular permeability and edema formation [18–20], while CGRP acts on CGRP1 receptors to dilate arterioles [21]. Both SP and CGRP released by activated afferent fibers promote the process of cutaneous EB extravasation. The sketch map of possible pathways is shown in Fig. 8.

### Mast cells were activated by nociceptive neuropeptides and then aggregated, degranulated, and released algogenic substances in EB dots

Mast cells are effector cells in allergic and anaphylactic reactions. However, during inflammatory responses mast cells can also respond to stimuli such as neuropeptides of SP, CGRP, independent of FceRI [22]. Tryptase is a neutral protease selectively concentrated in the secretory granules of mast cells (but not basophils), serving as a marker of mast-cell activation [10]. In this study mast cells in the skin of sensitized acupoints were activated to respond to neuropeptide SP and CGRP. They gathered, degranulated, and released 5-HT and HA in EB Dots (Fig. 8). Because of their anatomic association with cutaneous nerves, mast cells and their released substances appear to play an important role in mediating neuronal antidromic responses in the skin, although the precise role of these cells in cutaneous inflammation remains to be determined [23].

Previous studies showed that mast cells participated in the mechanism of analgesia induced by acupuncture [24, 25], moxibustion [26], and laser acupuncture [27]. Once mast cells are activated, the released mediators can be expected to activate sensory nerve fibers [23], and some of them, such as adenosine, have been suggested as a mediator of acupuncture-induced analgesia [28]. Further research is needed to investigate the role of mast cells in the effect of acupuncturing at sensitized acupoints.

### Revelation of paradoxes in acupoints research

Recently a number of well-designed clinical trials and systems reviews have reported that true acupuncture is superior to routine medication, but does not significantly outperform sham acupuncture. These findings are apparently at odds with traditional theories regarding acupuncture point specificity [29]. On the other hand, evidence from laboratory shows that stimulation of different points on the body causes distinct responses in hemodynamic, fMRI, and central neural electrophysiological responses [30]. These paradoxes in acupuncture research bring us a vital question. Is it reasonable to consider positions beside the acupoints as sham acupoints or nonacupoints?

This study sheds light on the paradox of acupoints. It indicates that the sensitized acupoints are limited to the related dermatomes and the location is not limited to a point because the area beside the EB point is also sensitized by increased expression of nociceptive substances. These results suggest that the areas beside EB dots are also activated, although less than the EB dots. Thus, sham acupoints beside the real acupoints are not an appropriate control.

## Conclusion

HCl-induced GMI results in neurogenic plasma extravasation in the skin of acupoints limited to T9-11 dermatomere. In the extravasated dots there is a biochemical milieu of substances associated with neuroinflammation, which includes elevated expressions of SP and CGRP in nerve fibers, and elevated expressions of HA and 5-HT in activated mast cells. The extravasated dots are an indication of sensitized acupoints, and are considered as an active response to the disease. The present study gives, for the first time, evidence of kinetic process of acupoints sensitization and its underappreciated mechanism. Further research is needed in regard to the effect of acupuncturing at sensitized acupoints.

## Abbreviations

GMI: Gastric mucosal injury
EB: Evans Blue
MCs: Mast cells
CGRP: Calcitonin gene-related peptide
SP: Substance P
HA: Histamine
5-HT: Serotonin
